# Survey of the Ciliary Motility Machinery of *Drosophila* Sperm and Ciliated Mechanosensory Neurons Reveals Unexpected Cell-Type Specific Variations: A Model for Motile Ciliopathies

**DOI:** 10.3389/fgene.2019.00024

**Published:** 2019-02-01

**Authors:** Petra zur Lage, Fay G. Newton, Andrew P. Jarman

**Affiliations:** Centre for Discovery Brain Sciences, Edinburgh Medical School, University of Edinburgh, Edinburgh, United Kingdom

**Keywords:** cilium, flagellum, *Drosophila*, ciliopathy, dynein

## Abstract

The motile cilium/flagellum is an ancient eukaryotic organelle. The molecular machinery of ciliary motility comprises a variety of cilium-specific dynein motor complexes along with other complexes that regulate their activity. Assembling the motors requires the function of dedicated “assembly factors” and transport processes. In humans, mutation of any one of at least 40 different genes encoding components of the motility apparatus causes Primary Ciliary Dyskinesia (PCD), a disease of defective ciliary motility. Recently, *Drosophila* has emerged as a model for motile cilia biology and motile ciliopathies. This is somewhat surprising as most *Drosophila* cells lack cilia, and motile cilia are confined to just two specialized cell types: the sperm flagellum with a 9+2 axoneme and the ciliated dendrite of auditory/proprioceptive (chordotonal, Ch) neurons with a 9+0 axoneme. To determine the utility of *Drosophila* as a model for motile cilia, we survey the *Drosophila* genome for ciliary motility gene homologs, and assess their expression and function. We find that the molecules of cilium motility are well conserved in *Drosophila*. Most are readily characterized by their restricted cell-type specific expression patterns and phenotypes. There are also striking differences between the two motile ciliated cell types. Notably, sperm and Ch neuron cilia express and require entirely different outer dynein arm variants—the first time this has been clearly established in any organism. These differences might reflect the specialized functions for motility in the two cilium types. Moreover, the Ch neuron cilia lack the critical two-headed inner arm dynein (I1/f) but surprisingly retain key regulatory proteins previously associated with it. This may have implications for other motile 9+0 cilia, including vertebrate embryonic nodal cilia required for left-right axis asymmetry. We discuss the possibility that cell-type specificity in ciliary motility machinery might occur in humans, and therefore underlie some of the phenotypic variation observed in PCD caused by different gene mutations. Our work lays the foundation for the increasing use of *Drosophila* as an excellent model for new motile ciliary gene discovery and validation, for understanding motile cilium function and assembly, as well as understanding the nature of genetic defects underlying human motile ciliopathies.

## Introduction

Motile cilia play important developmental and physiological roles concerned with the movement of fluid (e.g., airway cilia in mucociliary clearance, embryonic nodal cilia in left-right asymmetry determination) or movement through fluid (e.g., the sperm flagellum). Although many types of cilia are immotile and play a sensory role, the ancestral cilium is thought to have been motile, and the molecular machinery of ciliary motility is highly conserved. As such, a range of model organisms have been able to contribute much to our knowledge of motile cilia, from mammals to unicellular eukaryotes such as the biflagellate green alga, *Chlamydomonas reinhardtii* (King, 2016). From numerous studies over many years the structure and function of the motility apparatus is known in great detail, yet it is also bewilderingly complex with much remaining to be understood. In humans, the inherited disease, Primary Ciliary Dyskinesia (PCD) is caused by mutations in around 40 different genes encoding components of the motility apparatus. PCD is characterized by reduction or loss of ciliary motility, leading to defects in mucociliary clearance, organ left-right asymmetry, and male and female fertility (Mitchison and Valente, 2017). There is a continuing need to discover and validate new genes that may cause PCD when defective, as well as a need to understand the cellular and molecular functions of these genes. Model organisms can play a key role in advancing both these goals.

**Figure 1:**
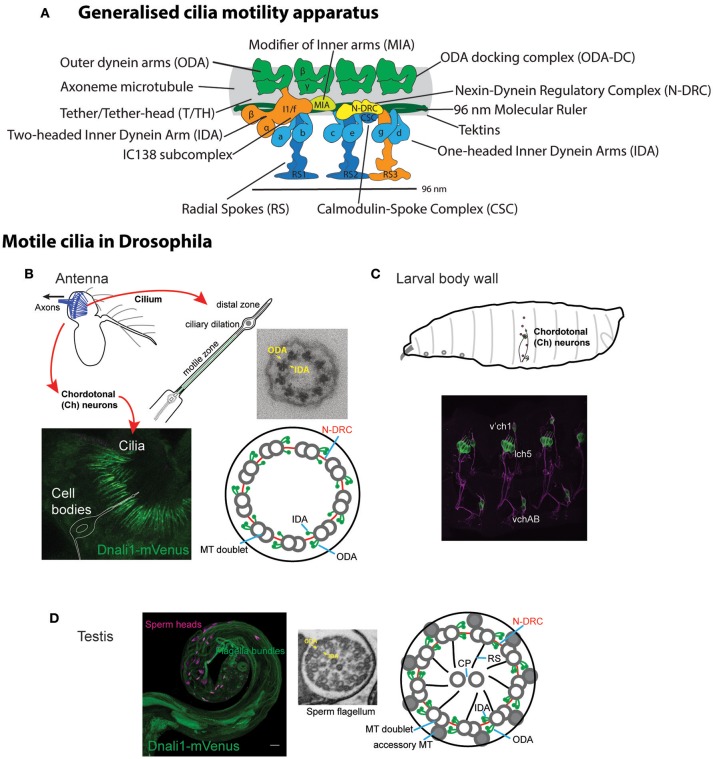
Motile ciliated/flagellated cells in *Drosophila*. **(A)** Schematic diagram of ciliary motility apparatus in a generalized motile cilium (based on cryoEM of human respiratory cilia in Lin et al., 2015). Center of cilium would be toward the bottom. Single repeat unit shown spanning 96 nm with 4 ODA complexes spaced every 24 nm, while the periodicity of different IDA forms is 96 nm. See Table 1 for glossary of terms. **(B,C)** *Drosophila* Ch neurons. **(B)** Auditory Ch neurons in the antenna. Each Ch neuron bears a terminal mechanosensory cilium with 9+0 axoneme structure. The proximal zone of the terminal cilium has dynein arms, marked in the image by *CG6971*-mVenus (*Dnali1*) (green). Ch neurons are also located in legs and wings (not shown), where they are proprioceptive. **(C)** Similar ciliated Ch neurons are present in the body wall of the larva, where they are both proprioceptive and auditory. **(D)** Sperm. Image shows sperm flagella bundles in testis marked with *CG6971*-mVenus (green), with sperm heads labeled with DAPI. In cross-section, the flagellum has a 9+2 axoneme structure and motile features. ODA, outer dynein arm; IDA, inner dynein arm; MT, microtubule; CP, central pair; RS, radial spoke, N-DRC, nexin-dynein regulatory complex.

The motors of ciliary movement are the outer and inner dynein arms (ODA, IDA, see Table 1 for abbreviations) that decorate the A tubules of the axonemal microtubule doublets (Figure 1A). These large multi-subunit complexes exist in several subtypes defined largely by their heavy chain (HC) constituents, which are likely required for different aspects of beat strength, frequency, and waveform (King, 2016). *Chlamydomonas* studies have shown that dynein activity is coordinated and modulated by several large “regulatory hub” complexes (Mitchell, 2017; Viswanadha et al., 2017; Porter, 2018), including: (a) the nexin–dynein regulatory complex (N-DRC), which both connects adjacent doublets and transmits information on interdoublet sliding (Bower et al., 2013); (b) the central pair-radial spoke (CP/RS) complex, which may transmit information between doublets to coordinate motor activity during bending to one side of axoneme only (Oda et al., 2014b); (c) the base of IDA subtype I1/f with the modifier of inner arms (MIA) complex, which may interface between the motors and the N-DRC/RS (Yamamoto et al., 2013). Several proteins are required for attachment (or docking) of these complexes along the axonemal microtubules in the correct periodicity. These include outer arm docking complex (ODA-DC) proteins and the “96 nm molecular ruler” proteins that guide spacing and attachment of IDA, RS, and N-DRC (Oda et al., 2014a). Tektins may also be required for IDA docking/attachment in addition to microtubule stability (Amos, 2008). As far as is known, the large variety of motile cilia in different organisms largely share this machinery, with the notable exception that some lack the CP/RS complexes, thereby having a 9+0 axonemal microtubule structure rather than 9+2. The most notable example of this exception is the nodal cilia of the vertebrate embryonic node that are required for left-right axis asymmetry.

**Table 1:** Glossary of motile cilia proteins and complexes.

**Name**	**Abbreviation**
Microtubule	MT
Outer dynein arm	ODA
Inner dynein arm	IDA
Two-headed inner dynein arm	IDA I1/f
Single-headed inner dynein arm	IDA a–g
Dynein heavy chain	HC
Dynein intermediate chain	IC
Dynein light chain	LC
Dynein light-intermediate chain	LIC
Nexin-dynein regulatory complex	N-DRC
Tether/tetherhead complex	T/TH
Modifier of inner arms	MIA
Calmodulin-spoke complex	CSC
Radial spoke complexes (1, 2, 3)	RS (RS1, RS2, RS3)
Central pair complex	CP
Outer dynein arm docking complex	ODA-DC
Intraflagellar transport	IFT
Dynein pre-assembly factor	DNAAF
A-kinase anchoring protein	AKAP

Construction of the motility machinery during ciliogenesis is itself an intricate process requiring specialized pathways of protein assembly and transport. The various motility complexes appear to be pre-assembled in the cytoplasm before being transported into the cilium as whole complexes (Fok et al., 1994; Fowkes and Mitchell, 1998; Viswanadha et al., 2014). The process is best known for ODA/IDA for which assembly requires a set of at least 11 dedicated proteins known as dynein assembly factors (DNAAFs) (Mitchison and Valente, 2017). At the base of the cilium, pre-assembled complexes are transported into the cilium by intraflagellar transport (IFT), requiring a specialized set of kinesin and dynein motors along with adaptor proteins.

Ecdysozoans such as nematodes and insects have lost cilia from almost all cells, with the remaining ciliated cell types having specialized roles. Similarly to *C. elegans*, the only somatic cells bearing cilia in *Drosophila* are the Type I sensory neurons, in which the cilium forms the terminal sensory apparatus and is the site of sensory transduction. Unlike *C. elegans*, however, some of these sensory neurons retain ciliary motility, namely the chordotonal (Ch) neurons that are required for proprioception and auditory reception. The 9+0 axoneme of the Ch neuron cilium is decorated with ODA/IDA in its proximal region (Figures 1B,C; Kavlie et al., 2010; Newton et al., 2012). Dynein activity is required for sensory mechanotransduction, probably as adaptation motors that drive active amplification and frequency tuning (Newton et al., 2012; Senthilan et al., 2012; Moore et al., 2013; Diggle et al., 2014; Karak et al., 2015). Apart from Ch neurons, the only other *Drosophila* cell type with axonemal motility is the spermatozoan, whose flagellum has a 9+2 structure (Figure 1D). The flagellum is unusually long (1.9 mm), and flagellogenesis is unusual in that it does not depend on IFT, and instead proceeds by a pathway of cytoplasmic construction followed by extrusion during sperm individualization (Han et al., 2003; Sarpal et al., 2003). Mutation of genes required for dynein structure or assembly results in viable flies with proprioceptive/auditory defects and male infertility (Kavlie et al., 2010; Moore et al., 2013). This is the fly equivalent of human PCD.

As might be expected, in *Drosophila* the expression of known ciliary motility genes is highly cell-type-specific (Cachero et al., 2011; Newton et al., 2012). Indeed in differentiating Ch neurons, motility gene transcription was found to be regulated by a combination of two transcription factors: the ciliogenesis regulator, Rfx, and the Foxj1-related factor, Fd3F (Laurençon et al., 2007; Newton et al., 2012); the paired Rfx/Fd3F binding sites form a motile cilium “gene regulatory code” (Cachero et al., 2011; zur Lage et al., 2011; Newton et al., 2012). This very restricted distribution provides benefits for transcriptomic screening and genetic analysis of candidate ciliary motility genes. This has recently enabled *Drosophila* to contribute to characterization of several novel dynein assembly factors, thereby aiding PCD gene discovery and validation (Moore et al., 2013; Diggle et al., 2014; zur Lage et al., 2018). However, the highly specialized nature of *Drosophila* motile cilia/flagella raises questions of how much of the ancestral motile cilium machinery is retained in this organism, and how these conserved aspects are distributed across the two motile ciliated cell types: 9+0/IFT-dependent and 9+2/IFT-independent.

Here, we comprehensively characterize the molecular basis of ciliary motility in *Drosophila*. Based on homology to genes required for ciliary motility in other organisms (particularly *Chlamydomonas* and human), we show that *Drosophila* has an almost full complement of ciliary motility genes, including orthologs of almost all human genes that have been associated with PCD. Based on transcriptome analysis, the majority of these genes are uniquely expressed in the two motile ciliated cell types. Genetic analysis by targeted RNA interference confirms that knockdown of many motility genes results in impaired motility that can be detected by simple proprioception and fertility analyses. Strikingly, our analysis reveals major differences in the expression of ciliary motors and related components between Ch neurons and spermatocytes. Notably, the two cell types harbor distinct ODA complexes and differ in the expression of a key IDA motor subtype (I1/f). Our analysis lays the basis for further use of *Drosophila* as a motile ciliopathy model, as well as for the understanding of cell-type specializations of motile cilia.

## Materials and Methods

### Gene Orthology

Eukaryotic ciliary motility genes were compiled from the literature, particularly of *Chlamydomonas* and human studies (Wickstead and Gull, 2007; King, 2016; Viswanadha et al., 2017). Orthology in *Drosophila* was assessed using DIOPT (Hu et al., 2011), with contributions from previous specific studies (Wickstead and Gull, 2007; Karak et al., 2015; Kollmar, 2016; Viswanadha et al., 2017; Neisch et al., 2018).

### Fly Lines

RNAi lines were obtained from Vienna *Drosophila* Resource Center (GD and KK lines) as shown in Table S1. Flies were maintained on standard cornmeal-agar medium at 25°C. *UAS-Dcr2; scaGal4. BamGal4* was provided by Helen White-Cooper (Cardiff University). *cato*-GFP is described in zur Lage and Jarman (2010).

### Transcriptome Analysis of Embryonic Ch Neurons

Transcriptome analysis was performed by fluoresence activated cell sorting (FACS) and microarray analysis using *cato*-GFP embryos similarly to previously described (Cachero et al., 2011). *cato*-GFP embryos were collected and aged at 25°C on grape juice agar plates. At 10:45–11:45 h age (stage 13/14 of embryonic development) embryos were dechorionated in 50% bleach for 2 min 30 s and washed thoroughly with water. The embryos were transferred to a Dounce homogeniser in dissociation medium [Shields and Sang M3 insect medium (Sigma) with 5% FBS (ThermoFisher)] and homogenized with 25 gentle strokes of a loose pestle avoiding foam formation. The cell suspension was then transferred to siliconised tubes previously rinsed in dissociation medium. After centrifugation at 1,000 g for 3 min at room temperature, the supernatant was discarded, and the pellet of cells was resuspended in 1 ml Trypsin (Sigma) in PBS. The suspension was incubated at room temperature for 7 min on a rotating wheel and after subsequent centrifugation at 1,000 g for 3 min at 4°C the supernatant was discarded. The cells were resuspended in 0.2 ml dissociation medium and transferred to a new tube containing 1 ml of dissociation buffer and centrifuged. This step was repeated once, before FACS was performed using a BD FACSAria cell sorter (Becton-Dickinson). The cells were collected into dissociation medium. Up to 300,000 GFP positive cells were sorted per tube and up to 1,000,000 GFP negative cells in a separate tube. Subsequently, the cells were spun at 1,000 g for 3 min at 4°C and the pellet was carefully resuspended in 300 μl of RLT lysis buffer (Qiagen) containing β-mercaptoethanol before storage at −80°C. mRNA from GFP-positive and -negative cells was hybridized to Affymetrix 2.0 microarrays by Glasgow Polyomics, University of Glasgow (3 replicates each). Differential expression was determined as a ratio of expression in GFP-positive vs. GFP-negative cells. Data analysis was performed in Partek Genomic Suite 6.6 with the dataset normalized using RMA normalization. One-way ANOVA was performed between positive and negative data with *P-*values then adjusted for multiple test correction using the FDR step-up method.

### *Drosophila* Embryo *in situ* Hybridization

Embryos were harvested from a 24 h collection at 25°C. After dechorionating the embryos in 50% chlorine bleach for 5 min, they were fixed for 20 min in 1:1 mixture of 3.7% formaldehyde/PBS and heptane while shaking at 200 rpm. After removing the PBS formaldehyde lower phase, methanol was added for devitellinisation. Settled embryos were collected and washed twice with methanol. Subsequently, the embryos were rehydrated in a stepwise manner starting with a 3:1, 1:1, and 1:3 ratio of methanol and PBST (PBS plus 0.1% Tween-20), before postfixing them in 3.7% formaldehyde for 20 min. To remove all traces of the latter, the embryos were washed 5 × 5 min in PBST, before incubating them in a 1:1 mixture of hybridization buffer (RNA hybridization buffer: 50% formaldehyde, 5x SSC, 50 μg/ml heparin, 100 μg/ml tRNA, 0.1% Tween-20, pH6.5). After another 10 min of incubation in hybridization buffer at room temperature, prehybridisation was performed for at least 2 h at 70°C in hybridization buffer. After removal of the prehybridisation buffer, the probe was added and incubation took place overnight. Wash solutions were heated at 70°C and after removing the probe, six washes were carried out for 30 min each using initially hybridization buffer, then a 1:1 ratio with PBST, followed by four PBST washes. After a brief wash with PBST at room temperature, the embryos were incubated for 2 h in a 1:2,000 dilution of anti-DIG-AP (alkaline phosphatase) (Roche) while rotating. After three 20 min washes in PBT, they were rinsed in reaction solution (100 mM Tris, pH9.5 and 100 mM NaCl). The color reaction was carried out following the NBT/BCIP protocol (Roche). Once the color reaction had sufficiently developed, the embryos were washed 3x in PBST to stop the reaction before being mounted on slides in 70% glycerol/PBS. Slides were imaged on an Olympus Provis AX-70 microscope using an UPlanApo 20x/0.7 objective. Images were cropped and adjusted for contrast in FIJI.

### RNA Probe Preparation

Antisense RNA probes were synthesized using the DIG RNA labeling kit (Roche) from a PCR product containing the T7 promoter on the right primer (Table S1). The DIG labeling reaction contained 100–200 ng purified PCR product in a final volume of 10 μl and was carried out for at least 2 h at 37°C. Subsequently, the probe was cleaned using the GeneJET RNA purification kit (ThermoFisher) and eluted in 30 μl of H_2_O. The probe was usually used in a 1:200 dilution in hybridization buffer after heating for 5 min at 95°C.

### RT-PCR Analysis

Total RNA was isolated from 120 pairs of adult antennae or 150 pairs of testes using the RNeasy Mini kit (Qiagen #74104). cDNA was synthesized using the ImProm-II™ Reverse Transcription system (Promega #A3800). Primers were designed to span at least one intron in order to distinguish mature mRNA from genomic DNA (Table S1). For genomic DNA control, DNA was isolated from adult flies using a standard *Drosophila* DNA extraction protocol. PCR amplification was carried out using Roche Taq polymerase (#4728874001).

### Genetic Analysis

RNAi knockdown was performed as described previously (zur Lage et al., 2018) using fly lines harboring inducible UAS-hairpin constructs. Knockdown was performed in sensory neurons (driven by *UAS-Dcr2; scaGal4*) or testes (*BamGal4*). For controls we used progeny from each Gal4 line crossed to the appropriate parent strain for the RNAi line in question. Locomotory coordination (requiring Ch neuron function) was tested in an adult climbing assay. Batches of 15 UAS*-Dcr2/*+*; scaGal4/*+*, UAS-RNAi/*+ flies were placed in a vertical sealed tube with gradations as 5, 10, and 15 cm. After banging down, flies were allowed to climb the tube for 30 s. At this point flies were scored according to their vertical location reached (1: <5 cm; 2: 5–10 cm; 3: 10–15 cm; 4: >15 cm). The average score constituted the Climbing Index for that batch (*n* = 5–10 batches per line). Average climbing index was transformed as a proportion of the climbing index achieved by the control flies (<1 represents defective climbing). Male fertility was tested by crossing individual UAS-RNAi/+; BamGal4/+ males (*n* = 10) to 2 OregonR females, allowing them to mate for 2 days, then transferring to new vials for two more days. The number of progeny (per male) from the latter vials were counted. In some cases, average number of progeny per male was determined and expressed as a ratio compared to the progeny produced by control males (<1 represents reduction in fertility). In cases where no or very few progeny were produced, infertility was recorded as the proportion of males yielding a complete lack of progeny. In some cases, production of motile sperm was assessed by examination of dissected and partially squashed testes by light microscopy. In all cases, significance was tested by ordinary 1-way ANOVA with Dunnetts *post-hoc* correction for multiple testing.

## Results

### Ciliary Motility Genes in *Drosophila*: Identification, Expression, and Genetic Requirement

Ciliary motility genes are well conserved among eukaryotes. To survey the presence of such genes in *Drosophila*, we compiled a gene list of eukaryotic ciliary motility genes derived particularly from *Chlamydomonas* and human studies (Wickstead and Gull, 2007; King, 2016; Viswanadha et al., 2017). Orthology in *Drosophila* was assessed primarily using the DRSC Integrative Orthology Prediction Tool (DIOPT) (Hu et al., 2011). We concentrated on genes that are required exclusively for the structure, function, or generation of motile cilia, thereby excluding structures (e.g., transition zone proteins) and processes (e.g., IFT complexes) shared with immotile or primary cilia. The orthologs identified are compiled in Tables 2–**4**, S2. In summary, we find that *Drosophila* has orthologs of almost all genes associated with ciliary motility. Among these genes are orthologs of almost all human genes that have been associated with PCD (**Table 6**).

**Table 2:** Axonemal dynein HC and IC genes in *Drosophila*.

**Gene**	**Name**	**Class/name**	***Chlamydomonas***	**Human**	**Dynein complex**	**Ch neuron expression**	**Testis expression**	**Expression summary**	**Expression corroboration**
**OUTER ARM DYNEIN**									
*CG45785*	*kl-3*	HC, DHC3A	OADgamma	*DNAH8*	ODA	(not on chip)	15.20 (38)	Ch + Testis	RT-PCR
*CG9492*		HC, DHC3B	OADgamma	*DNAH5*	ODA	3.13	4.50 (9.2)	Ch only	ISH, RT-PCR
*CG3723*	*Dhc93AB*	HC, DHC4A	OADbeta	*DNAH11*	ODA	8.12	0.20 (1.9)	Ch only	Newton et al., 2012
*CG45786*	*kl-5*	HC, DHC4B	OADbeta	*DNAH17*	ODA	(not on chip)	3.2 (13)	Testis only	
*CG3339*		HC, DHC4C	OADbeta	*DNAH9*	ODA	1.02	1.3 (6)	Ch only	RT-PCR
*CG9313*		IC, IC78	DIC1/ODA9	*DNAI1*	ODA	5.61	10.6 (1106)	Ch + Testis	ISH (Karak et al., 2015)
*CG6053*	*Dnai2*	IC, IC70	DIC2/ODA6	*DNAI2*	ODA	2.04	0.80 (3.1)	Ch only	
*CG10859*		IC, IC70	DIC2/ODA6	*DNAI2*	ODA	−2.13	13.70 (1580.2)	Testis only	
*CG1571*		IC, IC70	DIC2/ODA6	*DNAI2*	ODA	1.12	18.30 (246)	Testis only	
**INNER ARM DYNEIN, SINGLE-HEADED**									
*CG5526*	*Dhc36C*	HC, DHC7A	DHC6	*DNAH7*	IDA a,b,c,e	4.47	9.5 (288.1)	Ch + Testis	ISH
*CG17150*	*Dnah3*	HC, DHC7B	DHC5	*DNAH3*	IDA a,b,c,e	4.32	21 (391)	Ch + Testis	Karak et al., 2015
*CG15804*	*Dhc62B*	HC, DHC7C	DHC9	*DNAH12*	IDA a,b,c,e	8.34	8.4 (98.8)	Ch + Testis	Newton et al., 2012
No ortholog		HC, DHC8	DHC2	*DNAH1*	IDA d	na	na	na	
*CG7092*	*Dhc16F*	HC, DHC9A	DHC7	*DNAH6*	IDA g	3.85	8.7 (163.9)	Ch + Testis	ISH
No ortholog		HC, DHC9B	DHC3	*DNAH14*	IDA g	na	na	na	
*CG6971*		IC, LIC1	p28	*DNALI1*	IDA a,c,d	6.29	13 (707.9)	Ch + Testis	Newton et al., 2012
*CG31802*		IC, Centrin	DLE2	*CENTRIN*	IDA b,e,g	1.03	11 (1250.7)	Widely expressed	
**INNER ARM DYNEIN I1/F, TWO-HEADED**									
*CG1842*	*Dhc98D*	HC, DHC5	DHC1	*DNAH10*	IDA I1/f	1.18	14.30 (67)	Testis only	
*CG17866*	*kl-2*	HC, DHC6	DHC10	*DNAH2*	IDA I1/f	1.15	7.10 (54)	Testis only	
*CG14838*		IC, IC140	IC140/DIC3	*WDR63*	IDA I1/f	1.1	12.0, (112.9)	Testis only	
*CG13930*		IC, IC138	IC138/DIC4	*WDR78*	IDA I1/f	5.08	1.30 (4.5)	Ch only	Newton et al., 2012
*CG7051*	*Dic61B*	IC, IC138	IC138/DIC4	*WDR78*	IDA I1/f	−1.36	18.00 (256.3)	Testis only	
*CG15373*		IC, IC97	IC97	*CASC1/LAS1*	IDA I1/f,	1.12	1.7 (13.7)	Testis only	

*For HCs names, we follow the proposed nomenclature and relationships of Kollmar (2016). Ch expression: ratio of expression in differentiating Ch neurons from 12 h embryos relative to expression in rest of embryo. Testis expression: mRNA enrichment and expression signal (brackets) data from FlyAtlas. Expression summary: Ch expression (>2.0 differential expression) and testis expression (>1.5 enrichment, >10 signal). Expression corroboration: obtained from: ISH: mRNA in situ hybridization (this study), RT-PCR (this study), or from the reference cited. Where there is no ortholog, expression is not applicable (na)*.

For the identified orthologs, we characterized expression in the two motile ciliated cell types. For testis expression we utilized FlyAtlas adult tissue microarray data (Robinson et al., 2013). For Ch neurons, we determined the transcriptome of embryonic differentiating Ch neurons in a procedure similar to that which we previously reported (Cachero et al., 2011), but using later stage embryos in order to target terminal cellular differentiation and ciliogenesis. In short, we used a *cato*-GFP fly line, in which differentiating Ch neurons were marked by GFP expression (GFP is also expressed to a much lesser degree in other sensory neurons; zur Lage and Jarman, 2010). Embryos from timed egg collections were dissociated and GFP+ vs. GFP– cells were isolated by FACS. Gene expression was determined using Affymetrix 2.0 microarrays, and candidate Ch-enriched transcripts identified by ratio of expression in GFP+ cells vs. GFP– cells (i.e., the rest of the embryo) (data in Table S3). For selected genes, embryonic mRNA expression was confirmed by *in situ* hybridization or RT-PCR. In summary, the majority of orthologs were specifically expressed in one or both motile ciliated cell types (Tables 2–**4**), while a few were more widely expressed.

For further validation and to explore cell-type differences, the functional requirement of selected genes was tested by RNAi knockdown using UAS-hairpin lines. For Ch neuron function, flies with knockdown in sensory neurons (driven by *UAS-Dcr2; scaGal4*) were tested for defective locomotion in a climbing assay (which requires proprioceptive information from Ch neurons). For sperm function, male flies with testis-specific knockdown (driven by *BamGal4*) were tested their ability to produce offspring. Knockdown results are presented in **Table 5**.

In the following sections, we describe the *Drosophila* representation of ciliary motility genes and their expression starting with the dynein motors.

### *Drosophila* Has an Almost Complete Repertoire of Axonemal Dyneins

**Figure 2:**
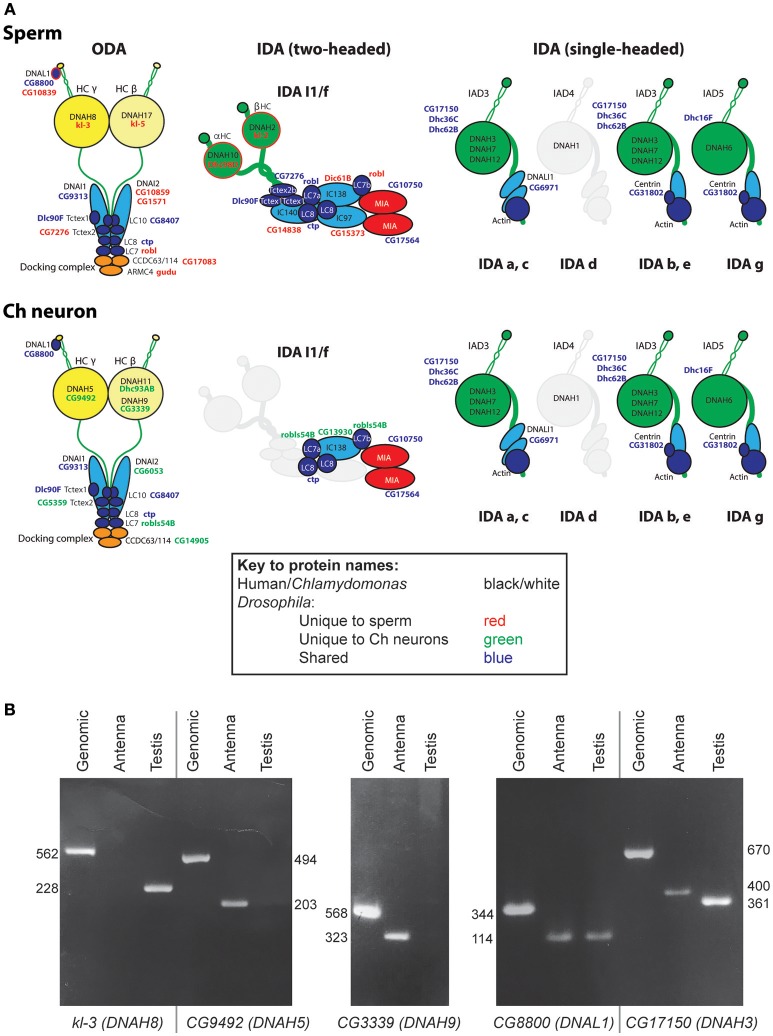
Axonemal dyneins in *Drosophila* cell types. **(A)** Summary of dynein subunits and subtypes represented in *Drosophila* as determined by homology and transcriptome analyses. The information is mapped onto schematics representing human or *Chlamydomonas* data (gene names in black or white), with corresponding *Drosophila* gene names in blue, red or green. Blue gene names are *Drosophila* subunits that appear in common across both motile ciliated cell types; red/green gene names are *Drosophila* subunits that appear unique to one or other cell type. Only form IDA d appears to be completely missing in *Drosophila* (grayed out). The ODA complexes have some shared subunits but also many that are unique to one or other cell type (including HCs, IC2, some LCs, docking complex chains). IDA I1/f motor is largely absent from Ch neurons except for a unique homolog of IC138 (CG13930) and potentially LC7 (robls54B). In contrast, the associated MIA subunits CG10750/CG17564 are present in both sperm and Ch neurons. The association of I1/f LCs is speculative as they are largely not unique to this motor. In contrast to these motors, monomeric IDA motors seem to be common between the cell types. **(B)** RT-PCR analysis of dynein HC expression in antenna and testis. Fragment lengths are shown in base pairs.

Dynein motors comprise ODAs docked at 24 nm intervals along the axoneme and several subtypes of IDA docked in a 96-nm repeating pattern (Figure 1A). Motor activity is largely performed by their Heavy Chains (HCs) while Intermediate Chains (ICs) often contribute to stable assembly of the complexes (Figure 2A). A variety of Light Chains (LCs) have accessory and regulatory functions. Whilst HCs and ICs are largely specific to each dynein subtype, many LCs are shared. In the account below, we concentrate on HCs, ICs, and those LCs that can be assigned to specific motor subtypes. Previous reports have identified several *Drosophila* homologs of axonemal dynein genes (Rasmusson et al., 1994; Newton et al., 2012; Karak et al., 2015). In our homology searches based on human and *Chlamydomonas* dynein chains (Wickstead and Gull, 2007; Hom et al., 2011; Kollmar, 2016; Viswanadha et al., 2017), we found that despite the specialized nature of its motile ciliated cells, *Drosophila* retains genes for an almost complete repertoire of ODAs and IDAs (Table 2; Figure 2A). In our analysis, we follow the dynein taxonomy proposed by Kollmar (2016).

### Outer Arm Dyneins: Different Forms in Ch Neurons and Sperm

In *Chlamydomonas*, ODA is thought to be the major motor for force generation (King, 2016). In metazoans, ODA is two-headed, containing HCs equivalent to beta and gamma HCs of *Chlamydomonas*. In humans, beta HCs are encoded by *DNAH9, DNAH11*, and *DNA17* while gamma HCs are encoded by *DNAH5* and *DNAH8* (unless otherwise stated, human gene designations are given hereafter). *Drosophila* has HC genes orthologous to each of these human chains (Kollmar, 2016; Table 2; Figure 2A). Interestingly, different gamma/beta gene pairs are exclusively expressed in each of the two cell types: *CG9492* and *Dhc93AB* in Ch neurons (corresponding to human *DNAH5/DNAH11*); *kl*-3 and *kl*-5 in sperm (corresponding to *DNAH8/DNAH17*). Consistent with this separation, both sperm-specific HCs are encoded by Y chromosome genes (Carvalho et al., 2000).

**Figure 3:**
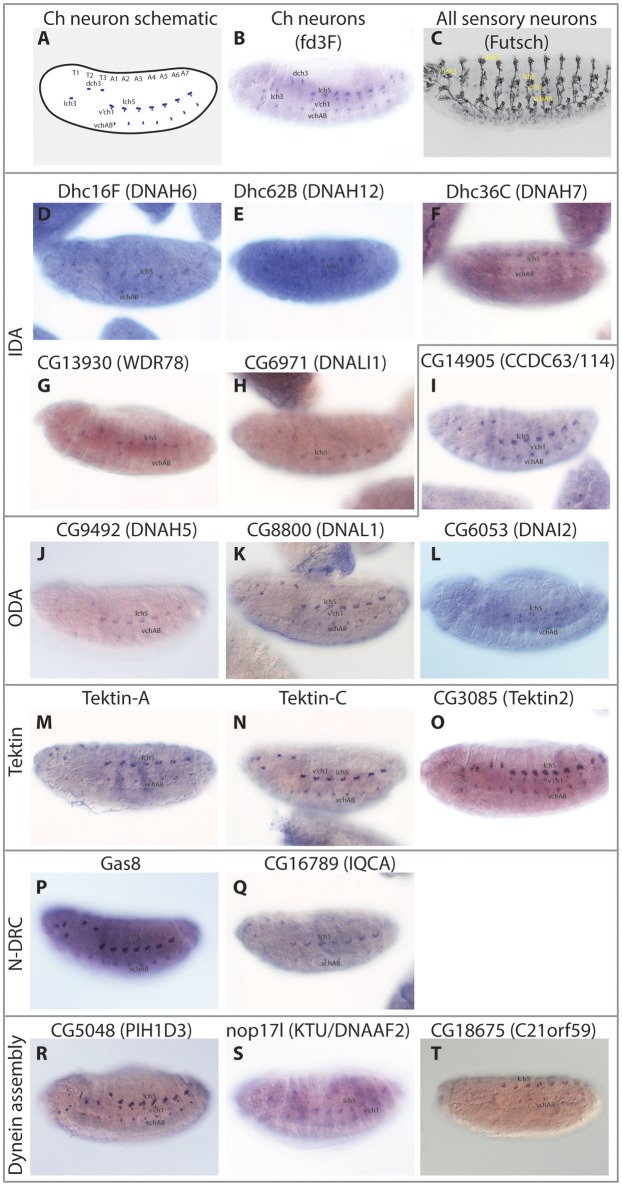
Embryonic expression patterns of ciliary motility genes. RNA *in situ* hybridisations in stage 14–16 embryos. **(A)** Schematic of late stage embryo showing the locations of Ch neurons. **(B)** For comparison, mRNA of *fd3F*, known to be uniquely expressed in differentiating Ch neurons (Newton et al., 2012). **(C)** For comparison, expression of Futsch protein in sensory neurons. This protein is expressed in both Ch neurons and other sensory neurons with immotile cilia. **(D–T)** The remaining panels show RNA expression of selected genes. In general, all are uniquely expressed or at least enriched (*nop17l*) in a pattern consistent with the distribution of Ch neurons.

To corroborate these differences, we analyzed gamma HC expression by RT-PCR. This confirmed that *kl*-3 expression is exclusive to testis while *CG9492* expression appears exclusive to antennae (which contain a large array of Ch neurons, Figures 1B, 2B). For comparison, an ODA LC (*CG8800/DNAL1*) was found to be expressed in both tissues (Figures 2A,B). *In situ* hybridization in embryos also confirms Ch-neuron specific expression of *Dhc93AB* and *CG9492*, although expression is quite weak, consistent with low transcription levels (Figures 3A–C,J; Newton et al., 2012). Further validation of cell-type specificity is provided by RNAi knockdown, which showed that the different ODA HCs yield phenotypes in only one or other cell type, thereby correlating with their expression patterns (**Table 5**).

A further beta HC (*CG3339*, corresponding to *DNAH9*), appears not to be highly expressed in either cell type by transcriptome analysis (Table 2). However, RT-PCR revealed that this HC is expressed in antennal Ch neurons (Figure 2B).

Human ODAs contain an IC heterodimer of DNAI1/DNAI2. *Drosophila* has a single *DNAI1* ortholog (*CG9313/Dnai1*), which is expressed in both motile ciliated cell types. RNAi knockdown confirms its requirement in both cell types (**Table 5**). In contrast, three *DNAI2* orthologs are present. One is Ch neuron-specific (*CG6053*) (Table 2; Figure 3L) and the other two are sperm-specific (*CG1571, CG10859*) (Figure 2A). RNAi knockdown of *CG6053* confirms that it is required for proprioception but not for male fertility (**Table 5**).

For LCs, *Drosophila* has homologs of all major families (Tctex1, Roadblock, and LC8 families) (Table S2). The distribution of LCs across dynein subtypes is not fully known, and indeed many subunits are not specific to axonemal dyneins. On current knowledge only DNAL1/LC1 is ODA-specific. *Drosophila* has two DNAL1 orthologs: as noted above, ortholog *CG8800* is expressed in both Ch neurons and testes, but *CG10839* is only expressed in testes (Figures 2A,B, 3K). For the Roadblock family (shared with IDA I1/f, below), several homologs are strongly expressed in testes, while *robls54B* is strongly enriched in Ch neurons.

**Figure 4:**
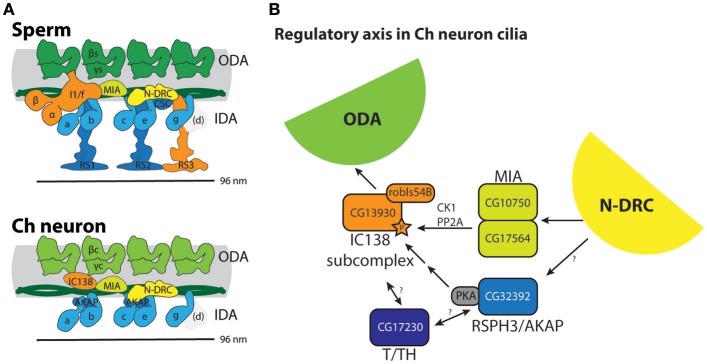
Summary of molecular apparatus for ciliary motility in *Drosophila*, as inferred from transcriptome and genetic analysis. **(A)** The predicted complexes are mapped onto schematic representations of human motility complexes based on cryoEM (Lin et al., 2015; Figure 1A). **(B)** Model for a regulatory axis in Ch neuron cilia, building on functional and biochemical models proposed for *Chlamydomonas* (Gaillard et al., 2006; Wirschell et al., 2011; Yamamoto et al., 2013; Fu et al., 2018). Surprisingly, despite the lack of CP/RS and functional IDA I1/f motor, Ch neuron cilia express several factors thought to transmit regulatory information from CP/RS to I1/f as well as ODA motors.

In conclusion, in *Drosophila* cell-type-specific ODA complexes exist characterized by different HCs, as well as some different ICs and LCs. This suggests divergence of ODA function in the two cell types (Figures 2A, 4A).

### Outer Dynein Arm Docking Complex (ODA-DC) Differs in Ch Neurons and Sperm

In *Chlamydomonas*, ODA-DC proteins are required to stabilize ODA docking on the axoneme (Oda et al., 2016). The human equivalent of ODA-DC is thought to consist of CCDC63, CCDC114 (DC2 homologs), and ARMC4; mutation of these genes causes PCD with loss of ODA (Hjeij et al., 2013; Knowles et al., 2013; Onoufriadis et al., 2013, 2014). In *Drosophila*, ODA-DC orthologs show strong differences in expression between sperm and Ch neurons (Table 3). Sperm and Ch neurons express distinct orthologs of CCDC63/114 (*CG17083* and *CG14905* respectively, Figure 3I). Knockdown of *CG14905* confirms a specific function in Ch neurons (**Table 5**). In contrast, the ortholog of ARMC4 (*CG5155/gudu*) is expressed only in sperm. Interestingly, human ARMC4 seems to be required for correct CP structure (Hjeij et al., 2013; Onoufriadis et al., 2014), and so lack of *gudu* expression in Ch neurons correlates with their lack of the CP.

**Table 3:** Other motility apparatus genes in *Drosophila*.

**Gene**	**Name**	***Chlamydomonas***	**Human**	**Ch neuron expression**	**Testis expression**	**Expression summary**	**Expression corroboration**
**MIA COMPLEX**							
*CG17564*		MIA2/FAP73	*CCDC43A/B*	78.21	12 (909)	Ch + Testis	
*CG10750*		MIA2/FAP73	*CCDC43B*	6.56	14 (783)	Ch + Testis	
No ortholog		MIA1/FAP100	*CCDC38*	na	na	na	
**T/TH COMPLEX**							
*CG17687*		CFAP43	*CFAP43*	3.2	204	Ch + Testis	
*CG34124*		CFAP44	*CFAP44*	Not on chip	Not on chip	Not detected	
**ODA DOCKING COMPLEX (ODA-DC)**							
*CG14905*		DCC2/ODA1	*CCDC63*	108.8	1.5 (6)	Ch only	ISH
*CG17083*		DCC2/ODA1	*CCDC114*	1.13	14 (980)	Testis only	
*CG5155*	*gudu*		*ARMC4*	1.07	11 (729)	Testis only	
**ODA ADAPTOR**							
*CG13202*			*CCDC103*	8.19	12 (257.3)	Ch + Testis	
**96 NM MOLECULAR RULER**							
*CG17387*			*CCDC39*	4.07	16 (316)	Ch + Testis	
*CG41265*	*l(2)41Ab*		*CCDC40*	4.06	14 (229)	Ch + Testis	
**PROTOFILAMENT STABILITY AND IDA DOCKING**							
*CG10541*	*Tektin-C*		*TEKT1*	3.6	15 (887)	Ch + Testis	ISH, Berkeley *Drosophila* Genome Project database
*CG4767*	*Tektin-A*		*TEKT4*	19.42	13 (1348)	Ch + Testis	ISH
*CG3085*			*TEKT2*	56.14	10 (1321)	Ch + Testis	ISH, Berkeley *Drosophila* Genome Project database
*CG17450*			*TEKT3/TEKT5*	3.69	14 (1054)	Ch + Testis	
**NEXIN-DYNEIN REGULATORY COMPLEX (N-DRC)**							
*CG10958*		DRC1	*DRC1/CCDC164*	4.81	12.1 (462)	Ch + Testis	Berkeley *Drosophila* Genome Project database
*CG30259*		DRC2	*CCDC65/CILD27*	1.67	0.9 (3)	(Ch only)	
*CG13125*	*TbCMF46*	DRC3	*LRRC48*	58.6	7.3 (89)	Ch + Testis	
*CG14271*	*Gas8*	DRC4	*GAS8*	3.2	12.5 (249)	Ch + Testis	ISH
*CG14325*		DRC5	*TCTE1*	1.7	0.2 (0)	(Ch only)	
*CG8272*		DRC6	*FBXL13*	1.2	1.5 (95)	Testis only	
*CG34110*	*lobo*	DRC7	*CCDC135*	34.2	11.1 (46)	Ch + Testis	
*CG11041*		DRC8	*EFCAB2*	9.9	0.4 (0)	Ch only	Berkeley *Drosophila* Genome Project database
*CG13972*		DRC9	*IQCG*	17.6	0.9 (0)	Ch only	
*CG13168*		DRC10	*IQCD*	1.99	13.6 (865)	Ch + Testis	
*CG16789*		DRC11	*IQCA*	30.7	1.6 (9)	Ch only	ISH, Berkeley *Drosophila* Genome Project database
**RADIAL SPOKE**							
*CG32392*		RSP3	*RSPH3/AKAP*	10.46	14 (801)	Ch + Testis	
*CG5458*		RSP1	*RSPH1*	1.03	14 (826)	Testis only	
*CG31803*			*RSPH9*	−1	11 (1011)	Testis only	
*CG3121*		RSP4/6	*RSPH4A*	1.08	16 (695)	Testis only	
*CG2981*	*TpnC41C*	RSP7	*CALML5*	−1.53	not detected	not detected	
*CG10014*		RSP11	*ROPN1L*	1.1	14 (811)	Testis only	
*CG17266*		RSP12	*PPIL6*	−1.45	0.6 (64)	Testis only	
*CG8336*		RSP12	*PPIL6*	1.4	3.5 (388)	Testis only	
*CG13501*		RSP14	*RTDR1*	−1.01	12 (278)	Testis only	
*CG10578*	*Dnaj-1*	RSP16	*DNAJB13*	1.64	2.0 (1378)	Widely expressed	
*CG15547*		RSP23	*NME5*	1.4	8.9 (242)	Testis only	
**CALMODULIN-SPOKE COMPLEX (CSC)**							
*CG30275*		FAP61	*C20orf26*	1	17 (396)	Testis only	
*CG30268*		FAP61	*C20orf26*	1	16 (254)	Testis only	
*CG15143*		FAP91/CaM-IP2	*MAATS1*	1.62	14 (158)	Testis only	
*CG15144*		FAP91/CaM-IP2	*MAATS1*	−1.05	14 (334)	Testis only	
*CG15145*		FAP91/CaM-IP2	*MAATS1*	−1.13	19 (284)	Testis only	
No ortholog		FAP251	*WDR66*	na	na	na	
**OTHER**							
*CG17230*			*CCDC11/CFAP53*	3.8	820	Ch + Testis	
No ortholog			*HYDIN*	na	na	na	

*Columns and abbreviations as for Table 2. CG17450 seems to be triplicated in Drosophila melanogaster (with CG32820, CG32819)*.

In humans, another protein, CCDC103, is independently required for ODA docking (Panizzi et al., 2012; King and Patel-King, 2015). The *Drosophila* ortholog (*CG13202*) differs from human CCDC103 in not possessing an RPAP3 domain, but it is nevertheless expressed in Ch neurons and testes. Knockdown of *CG13202* confirms at least a partial requirement in sperm and Ch neurons (**Table 5**).

### Two-Headed Inner Arm Dynein I1/f: Present in Sperm but Lacking From Ch Neurons

IDA I1/f is thought to regulate movement by resisting other dyneins. It is two-headed with two HCs and three ICs. *Drosophila* genes characteristic of this complex include homologs of alpha and beta HC genes (DNAH10: *Dhc98D* and DNAH2: *kl-2*), and the three ICs (WDR78/IC138: *Dic61B*; WDR63/IC140: *CG14838*; LAS1/IC97: *CG15373*) (Table 2). Each of these genes is expressed in testes, suggesting that sperm have a functional I1/f motor and associated machinery (Figure 2A). In striking contrast, none of these sperm HCs and ICs are expressed in Ch neurons, which therefore lack the I1/f motor. RNAi knockdown of the I1/f HCs confirms that this subtype has a role in sperm but not in Ch neurons (**Table 5**). This is corroborated by male infertility of *Dic61B* mutants (Fatima, 2011). In *Chlamydomonas* it has been suggested that this dynein is intimately involved in receiving and responding to regulatory signals from the CP/RS system (Viswanadha et al., 2017). Thus, *Drosophila* seems to support this role in that the absence of functional IDA I1/f from Ch neurons correlates with their lack of CP/RS (Figure 4A).

### Ch Neurons Retain Key Regulatory Subunits Associated With I1/f Dynein and Radial Spokes

In contradiction to their lack of HC expression for IDA I1/f, we found that Ch neurons surprisingly uniquely express *CG13930*, a second ortholog of the I1/f subunit, WDR78/IC138 (Figures 2A, 3G). Although this is an IC of I1/f, it is also thought to form part of an “IC138 subcomplex” that mediates regulation of motor activity by signals emanating from the RS and CP (Viswanadha et al., 2017; Figure 1A). Knockdown of *CG13930* confirms that it is required in Ch neurons but not in testes, despite the lack of I1/f motor and CP/RS in Ch neurons (**Table 5**). In Ch neurons, *CG13930* is potentially in a subcomplex with several LCs including *robls54B*, but this is not certain because no LCs are specific to dynein I1/f (Figure 2A; Table S2).

Given this finding, we examined expression of other proteins thought to interact with IDA I1/f. The MIA complex is proposed mediate transmission of signals from CP/RS via phosphorylation of IC138 (Yamamoto et al., 2013; Figure 1A). *Drosophila* has two orthologs of MIA2/FAP73 (*CG17564* and *CG10750*), and these are expressed in both testes and Ch neurons (Table 3; Figure 2A). The MIA transcripts are particularly highly enriched in Ch neurons (Tables 3, S1). In *Chlamydomonas*, IDA I1/f HCs are also associated with and regulated by a “tether and tether-head” (T/TH) complex (CFAP43/CFAP44). Unexpectedly, the *Drosophila* homolog of CFAP43 (*CG17687*) is expressed not only in testes, but also in Ch neurons. A CFAP44 homolog also exists (*CG34124*) but it is not represented on the microarray used in our expression analysis.

Given that these regulatory subunits are thought to link to CP/RS function, we analyzed the presence and expression of RS proteins. These are not well characterized in metazoans, but many RS proteins of *Chlamydomonas* have orthologs in *Drosophila* (Table 3). As expected, most are expressed in testes and not in Ch neurons, confirming that the latter's 9+0 cilia lack RS complexes. However, the RSPH3 homolog, *CG32392*, is an interesting exception as it is highly expressed in both cell types (Table 3). Interestingly, although RSPH3 is an RS component, it is predicted to act as an A-kinase anchoring protein (AKAP) to regulate the IC138 phospho-regulator. *CG32392* may therefore have an RS-independent function linked to a Ch neuron-specific IC138/*CG13930* regulatory complex.

Radial spokes are associated with a Calmodulin and Spoke-associated Complex (CSC), which is required for RS2 assembly and modulating dynein activity, possibly by transmitting signals from the RS to dyneins via N-DRC (Dymek et al., 2011). CSC subunits FAP61 and FAP91 have *Drosophila* homologs that expressed in testes, but none are enriched in Ch neurons.

In conclusion, despite their lack of CP/RS complex and functional I1/f dynein, Ch neurons express several key proteins previously associated with transmitting signals from CP/RS to dynein motors (Figures 2A, 4A), suggesting their cilia retain key parts of this regulatory axis for another purpose.

### Single-Headed Inner Arm Dyneins: Shared in Both Sperm and Ch Neurons

Six single-headed dynein variants are thought to exist in *Chlamydomonas* and humans (a–e, g), as classified by their HC and IC constituents. These IDA forms are thought to function as dyad pairs with one DNALI1/p28-containing and one centrin-containing motor in each dyad—a/b, c/e, g/d (Hirose and Amos, 2012; Figure 1A). *Drosophila* has four HC genes that encode orthologs of chains found in IDA a, b, c, e, and g (Wickstead and Gull, 2007; Table 2; Figure 2A). However, no d-specific HC gene (ortholog of human *DNAH1*) is present in *Drosophila*. Curiously, however, *DNAH1* has an apparent ortholog in *Apis mellifera* (Kollmar, 2016). Single-headed IDAs contain either centrin (b, e, d) or DNALI1/p28 (a, c, g) (Figure 2A). In *Drosophila*, the DNALI1 ortholog, CG6971, is localized to Ch neuron cilia and sperm flagella (Figure 3H; Diggle et al., 2014).

In contrast to the other dyneins, none of the single-headed IDA HCs are unique to one or other motile ciliated cell type in *Drosophila*: all are expressed in Ch neurons and testes (Table 2; Figures 2A, 3D–F). RT-PCR analysis of *CG17150* (*DNAH3*) confirmed that it is expressed in both antennae and testes. Interestingly, however, this analysis also revealed that the antennal transcript includes an extra penultimate 39-bp exon compared to testis (and not annotated in the *Drosophila* genome) (Figure 2B). Knockdown of *Dhc16F, Dhc36C*, and *Dhc62B* all result in full or partial reduction of fertility and climbing (**Table 5**).

### Attachment of Inner Dynein Arms (the “Molecular Ruler” and Tektins)

In humans, CCDC39 and CCDC40 proteins associate, and mutation of either can cause PCD with loss of N-DRC, RS, and DNALI1-containing IDAs (Becker-Heck et al., 2011; Merveille et al., 2011; Antony et al., 2013). Work in *Chlamydomonas* suggests that an elongated CCDC39/40 complex (FAP59/FAP172) forms a 96 nm molecular ruler to guide periodicity of docking of RS, N-DRC, IDAs (Oda et al., 2014a; Figure 1A). *Drosophila* has orthologs of both genes (*CG17387* and *l(2)41Ab*) and each is expressed in Ch neurons and testes (Table 3). RNAi knockdown of *CG17387/CCDC39* causes a severe climbing phenotype as well as immotile sperm (**Table 5**).

Tektins appear to be required for IDA assembly on, or attachment to, the microtubule doublets, but it is not clear whether they also have other functions in both motile and non-motile cilia (Tanaka et al., 2004; Amos, 2008; Linck et al., 2014). *Drosophila* has four tektin genes, all of which are expressed in testes and enriched in Ch neurons. *In situ* hybridization confirmed that *Drosophila Tektin*-A, *Tektin*-C and *CG3085* are expressed in Ch neurons, but they are not expressed in sensory neurons with non-motile cilia (Figures 3M–O). Knockdown shows that these tektins have some function in fertility, but Ch neuron phenotypes are not observed, perhaps due to redundancy or compensation (**Table 5**).

### Nexin–Dynein Regulatory Complex (N-DRC) Has Cell-Type-Specific Subunits

N-DRC is a large complex that bridges the microtubule doublets in motile cilia (Figure 1A). In *Chlamydomonas* it has at least 11 subunits and helps to maintain axonemal alignment and resistance to sliding (Bower et al., 2013). It is also an important regulatory hub that contacts ODA, IDA I1/f, and RS2 (Viswanadha et al., 2017). Human homologs of some subunits cause PCD with only subtle defects of ciliary beating [DRC4/*GAS8* (Jeanson et al., 2016; Lewis et al., 2016), *DRC1*/*CCDC164* (Wirschell et al., 2013), *CCDC65* (Horani et al., 2013)] while in *Chlamydomonas, drc* mutants still retain 9+2 structure and motility (Bower et al., 2013). In *Drosophila*, orthologs of most human N-DRC subunits can be identified, and many are expressed in both Ch neurons and testis (Table 3). *Gas8* and *CG17689* expression in Ch neurons was confirmed by *in situ* hybridization (Figures 3P,Q). Despite subtle effects of mutation in N-DRC genes in human and *Chlamydomonas* cilium beating, knockdown of *Drosophila Gas8* and *CG10958* (*CCDC164*/*DRC1*) clearly impairs climbing and male fertility (**Table 5**). The expression of N-DRC components in Ch neurons but not in other sensory neurons that have non-motile cilia (Figures 3P,Q) supports the hypothesis that this complex is structurally and functionally important in both 9+2 and 9+0 motile cilia but not responsible for axoneme stability in non-motile cilia (Porter, 2018). Surprisingly homologs of some components that might be considered core subunits are expressed exclusively in Ch neurons or in testes, suggesting cell-type specific variations in N-DRC (Table 3). Notably, *CG30259* (*CCDC65/DRC2*) appears to be absent from sperm. Consistent with this, knockdown of *CG30259* only affects Ch neuron function (**Table 5**).

### Motile Ciliogenesis: ODA Late Assembly/Transport Genes Are Expressed Exclusively in Ch Neurons

In *Chlamydomonas*, ODA5 and ODA10 are related to ODA-DC proteins, but are thought to be required along with ODA8 for maturation of pre-assembled ODA complexes prior to association with IFT machinery (Mitchell, 2017). Human ODA10 homolog *CCDC151* is mutated in PCD, and is required for stable localization of ODA-DC proteins (CCDC114 and ARMC4) in human and zebrafish (Hjeij et al., 2014; Jerber et al., 2014). In *Drosophila, CG14127* (*CCDC151*) and *CG14185* (*ODA8/LRRC56*) are both expressed exclusively in Ch neurons, thus correlating with the only motile ciliated cell type that requires IFT (Table 4). As noted by Jerber et al. (2014), this supports a role in linking dynein complexes to IFT for transport through the cilium. In addition, human PCD-associated *TTC25* is also proposed to link *CCDC151* to IFT (Wallmeier et al., 2016). Consistent with this, the *Drosophila* ortholog *CG13502* is exclusively expressed in Ch neurons (Table 4).

**Table 4:** Genes for dynein motor assembly and transport in *Drosophila*.

**Gene**	**Name**	***Chlamydomonas***	**Human**	**Ch neuron expression**	**Testis expression**	**Expression summary**	**Expression corroboration**
**ODA LATE ASSEMBLY COMPLEX**							
*CG14127*		ODA10/ODA5	*CCDC151*	2.22	not detected	Ch only	
*CG14185*		ODA8	*LRRC56*	2.46	not detected	Ch only	
*CG13502*			*TTC25*	3.48	1 (9.2)	Ch only	
**DYNEIN CYTOPLASMIC PREASSEMBLY FACTORS**							
*CG31623*	*dtr*	ODA7	*DNAAF1/LRRC50*	2.38	16 (648)	Ch + Testis	
*CG1553*	*nop17l*	PF13?	*DNAAF2/KTU*	1.20	0.3 (74)	Widely expressed	ISH
*CG5048*		PF13?	*PIH1D3*	55.39	9.7 (1891)	Ch + Testis	ISH, Berkeley *Drosophila* Genome Project database
*CG17669*		PF22	*DNAAF3*	19.61	14 (347)	Ch + Testis	ISH, Berkeley *Drosophila* Genome Project database
*CG14921*		PF23	*DNAAF4/DYX1C1*	5.20	8.9 (294)	Ch + Testis	ISH, Berkeley *Drosophila* Genome Project database
*CG14620*	*tilB*		*LRRC6*	9.40	12 (191)	Ch + Testis	
*CG11253*	*Zmynd10*		*ZMYND10*	41.98	12 (503)	Ch + Testis	ISH (Newton et al., 2012)
*CG31320*	*Heatr2*		*HEATR2/DNAAF5*	12.26	7.8 (493)	Ch + Testis	ISH, Berkeley *Drosophila* Genome Project database (Newton et al., 2012)
*CG18675*			*C21orf59*	52.13	15 (871)	Ch + Testis	ISH
*CG18472*			*SPAG1*	17.31	12 (410)	Ch + Testis	ISH (zur Lage et al., 2018)
*CG9750*	*reptin*		*REPTIN/RUVBL2*	2.53	2.6 (700)	Widely expressed	ISH, Berkeley *Drosophila* Genome Project database
*CG4003*	*pontin*		*PONTIN/RUVBL1*	1.59	2.1 (477)	Widely expressed	ISH (zur Lage et al., 2018)
*CG14353*	*Wdr92*		*WDR92*	4.10	6.3 (376)	Ch + Testis	ISH (zur Lage et al., 2018)

*Columns and abbreviations as for Table 2*.

### Motile Ciliogenesis: Dynein Assembly Factors Are Shared Between Ch Neurons and Sperm

A cohort of some 11 proteins, largely identified through characterization of PCD mutations, are required for the cytoplasmic pre-assembly of axonemal dynein complexes (Mitchison and Valente, 2017). All known DNAAFs have clear orthologs in *Drosophila* (Table 4). Despite the cell-type-specific differences in motors, these orthologs are highly expressed in both testes and Ch neurons (Table 4; Figures 3R–T), and have clear defects in both cell types upon knock-down (Table 5).

**Table 5:** Effects of gene knock down on ciliary motility functions.

***Drosophila***	**Human**	**Fertility relative to wild type**	***P-*value for fertility**	**Climbing index relative to wild type**	***P-*value for climbing**	**Phenotype summary**	**Note**
*Dhc93AB*	*DNAH11*	1.07	ns	0.62	<0.0001	Ch only	KK
*CG9492*	*DNAH5*	0/10 infertile		0.81	0.0096	Ch only	KK
*Dhc16F*	*DNAH6*	10/10 infertile		0.73	<0.0001	Ch + Testis	GD
*Dhc62B*	*DNAH12*	3/5 infertile		0.62	<0.0001	Ch + Testis	KK
*Dhc36C*	*DNAH7*	0.43	<0.0001	0.67	<0.0001	Ch + Testis	GD
*Dhc98D*	*DNAH10*	9/10 infertile		1.01	ns	Testis only	KK
*CG13930*	*WDR78/IC138*	1.19	ns	0.83	0.050	Ch only	GD
*CG9313*	*DNAI1*	10/10 infertile		0.63	<0.0001	Ch + Testis	KK
*CG6053*	*DNAI2*	0.95	ns	0.90	<0.0001	Ch only	KK
*CG6971*	*DNALI1*	9/10 infertile		0.83	0.0126	Ch + Testis	KK
*CG14905*	*CCDC63/114*	1.15	ns	0.59	<0.0001	Ch only	KK
*Tektin-C*	*TEKT1*	0.28	<0.0001	1.07	ns	Testis only	KK
*Tektin-A*	*TEKT4*	0.51	0.02	0.99	ns	Testis only	KK
*CG3085*	*TEKT2*	0.72	ns	0.99	ns	None	KK
*CG14127*	*CCDC151*	1.23	ns	0.81		Ch only	GD
*CG13202*	*CCDC103*	5/5 infertile		0.77	0.0002	Ch + Testis	KK
*CG10958*	*CCDC164*	9/10 infertile		0.64	<0.0001	Ch + Testis	KK
*CG30259*	*CCDC65*	1/5 infertile		0.73	<0.0001	Ch only	KK
*Gas8*	*GAS8*	5/5 infertile		0.65	<0.0001	Ch + Testis	KK
*CG17387*	*CCDC39*	0.07	<0.0001	0.62	<0.0001	Ch + Testis	KK
*CG17564*	*MIA2*	0.88	ns	0.81	<0.0001	Ch only	GD
*dtr*	*DNAAF1*	10/10 infertile		0.56	<0.0001	Ch + Testis	GD
*nop17l*	*DNAAF2*	0.08	<0.0001	0.72	<0.0001	Ch + Testis	GD
*CG5048*	*PIH1D3*	0.42	<0.0001	0.65	<0.0001	Ch + Testis	GD
*CG17669*	*DNAAF3*	0.50	0.0002	0.54	<0.0001	Ch + Testis	GD
*CG14921*	*DNAAF4*	0.31	<0.0001	0.87	0.0013	Ch + Testis	KK
*tilB*	*LRRC6*	10/10 infertile		0.44	<0.0001	Ch + Testis	KK (Kavlie et al., 2010)
*Zmynd10*	*ZMYND10*	Immotile sperm		Defective		Ch + Testis	Moore et al., 2013
*Heatr2*	*HEATR2*	Immotile sperm		Defective		Ch + Testis	Diggle et al., 2014
*reptin*	*REPTIN*	Immotile sperm		Lethal		?Ch + Testis	zur Lage et al., 2018
*pontin*	*PONTIN*	Immotile sperm		Lethal		?Ch + Testis	zur Lage et al., 2018
*CG18675*	*C21orf59*	10/10 infertile		0.60	<0.0001	Ch + Testis	KK
*CG18472*	*SPAG1*	0.20	<0.0001	0.49	<0.0001	Ch + Testis	KK
*Wdr92*	*WDR92*	Immotile sperm		Defective		Ch + Testis	zur Lage et al., 2018

*Fertility phenotype is summarized as number of progeny per male relative to control or as the number of completely infertile males. Proprioceptive phenotype is summarized as climbing assay index (height climbed) relative to control (therefore an index of 1 means no reduction). ns, not significantly decreased. RNAi lines used are from the GD and KK collections of the Vienna Drosophila Resource Center (Table S1). P-values are shown where a difference from control reaches significance (≤0.05) in ordinary 1-way ANOVA with Dunnetts post-hoc correction*.

Several PIH (Protein Interacting with Hsp90) domain proteins are involved in dynein assembly (Omran et al., 2008; Dong et al., 2014; Olcese et al., 2017; Paff et al., 2017), and *Drosophila* has functional homologs of each of these (Tables 4, 5). Human *PIH1D3* and *DNAAF2/KTU* are both PCD-causative genes (Omran et al., 2008; Olcese et al., 2017; Paff et al., 2017). *PIH1D3* is represented in *Drosophila* by *CG5048*. This gene is highly expressed in Ch neurons and testes, consistent with a specific role in dynein assembly (Table 4; Figure 3R). In contrast *CG1553/nop17l* (the ortholog of *DNAAF2/KTU*) is expressed only moderately in testes and its expression is not specific to that tissue in adults (Table 4). It is not strongly enriched in Ch neurons either, with RNA *in situ* hybridization revealing widespread expression, but somewhat elevated in Ch neurons (Figure 3S). It seems likely that *nop17l* is not dedicated solely to dynein assembly in *Drosophila*.

## Discussion

We found in general that the entire ciliary motility apparatus is highly conserved in *Drosophila* (Figure 4A). This suggests that despite the restricted distribution and function of its motile cilia, *Drosophila* has great potential as a genetic model for metazoan motile cilia biology. Indeed, the fact that only two cell types have motile cilia can be turned to advantage: it aids gene discovery and characterization, and it also provides a simple model system for exploring how diversity of motile cilium structure and function might be explained by cell-type-specific differences in ciliary motility machinery. Our cell type-specific comparison is illuminating: we find interesting differences in expression of dynein motors and other components that can be related to differences in ciliary structure (9+2 vs. 9+0), function, and mode of ciliogenesis (IFT vs. cytoplasmic assembly) (Figure 4A).

### Cell Type-Specific Differences in Axonemal Motors

For the dynein motor complexes, all expected metazoan outer and inner dynein subtypes are represented in the *Drosophila* genome except for the IDA d subtype (containing the DNAH1 HC in humans). Human *DNAH1* function (but not expression) appears restricted to sperm (Ben Khelifa et al., 2014). Single-headed IDAs work in pairs (dyads). In the absence of IDA d in *Drosophila*, it seems likely that this position on the axoneme is filled by another IDA form in order to complete the last IDA dyad (Figures 2A, 4A).

Our analysis has revealed interesting differences between the two motile ciliated cell types. Notably, their ODA motors appear entirely distinct, being distinguished by different HC pairs, different IC DNAI2 variants and a sperm-specific LC DNAL1 variant (Figures 2A, 4A). ODA motor differences presumably reflect the different functions of ciliary motility between the two cell types. In sperm, the motors must generate substantial force at a relatively low frequency for movement. In Ch neuron cilia, motors are required for the highly specialized process of auditory mechanotransduction, putatively for active mechanical amplification and probably also for sensory adaptation. In hearing, the whole antenna “quivers” in response to sound and it also generates spontaneous movements in the same frequency range in the absence of sound. It is known that these characteristics require axonemal dynein motor function within the Ch neurons (Göpfert and Robert, 2003; Newton et al., 2012; Karak et al., 2015), but the movement of Ch neuron cilia has not thus far been directly investigated. However, the Ch cilium motors must potentially respond with high temporal resolution (e.g., antennal Ch neurons are tuned to 100–300 Hz auditory stimuli; cf the ciliary beat frequency of human respiratory cilia of c.15 Hz). We propose that the Ch neuron ODA HCs (*CG9492* and *Dhc93AB*) are force generating for adaptation and amplification in mechanotransduction. Here we found that knockdown of either HC results in defective proprioception, consistent with defective Ch neuron mechanotransduction.

A second striking difference between the cell types is the presence in sperm but not Ch neuron cilia of a functional two-headed IDA I1/f dynein motor (based on the expression and genetic requirement of its HCs). In *Chlamydomonas* this dynein is thought to be important for regulating the size and shape of the axonemal bend, but clearly this motor function is dispensable in Ch neuron cilia.

In striking contrast to ODAs and IDA I1/f, Ch neurons and sperm appear to share the same single-headed IDA dynein subtypes. The DNAH3 homolog (CG17150) is required for hearing and male fertility (Karak et al., 2015) and we found that *Dhc16F, Dhc36C*, and *Dhc62B* are also required for normal proprioception and male fertility. Even for these single-headed IDAs, however, our limited expression analysis suggests that cell-type specific variations exist: we found that CG17150/DNAH3 exists as a different isoform in Ch neurons vs. sperm as a result of alternative splicing (Figure 2B). Therefore, dynein motor HCs vary between different cell types both in terms of presence/absence and also by the presence of cell-type specific isoforms.

### Other Cell Type Differences: Differences in Function or Differences in Mode of Ciliogenesis?

Some cell type differences in motility proteins can be ascribed to their requirement for IFT during ciliogenesis, and therefore are not expressed in IFT-independent sperm. This includes the ODA late assembly proteins (*CG14127, CG14185*) and the *TTC25* homolog (*CG13502*), all of which are Ch neuron-specific and have previously been linked to IFT (Jerber et al., 2014). In addition, there are strong differences in ODA-DC proteins expressed in each cell type. This may reflect the different docking requirements of the different ODA motors in each cell type, or the need to interact with IFT machinery in Ch neuron ciliogenesis.

Interestingly, we find some differences in expression of N-DRC subunits between cell types: for example, the *DRC2* homolog, *CG30259* appears to be expressed and required only in Ch neurons. Whether this reflects cell type differences in N-DRC function or transport remains to be determined. N-DRC structure and function is only beginning to be understood in *Chlamydomonas* (Porter, 2018), and is very poorly known in other organisms. However, it has been noted that the *Chlamydomonas* homolog of *DRC2* is required for N-DRC assembly, perhaps due to its association with IFT? We suggest that *Drosophila* will be a useful model for exploring the possibility of cell type-specific differences in N-DRC.

Conversely, the observation that dynein pre-assembly factor (DNAAF) homologs are all required in both motile ciliated cell types corroborates the view that they are required for cytoplasmic pre-assembly of motors rather than for their IFT-dependent trafficking.

### An Unusual Motor Regulatory Axis in Ch Neuron Cilia

In *Chlamydomonas*, part of the distinctive IDA I1/f motor forms the “IC138 regulatory subcomplex,” which is thought to control motor activity and microtubule sliding based on signals transmitted from the CP/RS complex and N-DRC via the MIA complex (Figure 1A; Bower et al., 2009; Yamamoto et al., 2013; Hwang et al., 2018). Regulation of IC138 is partly through phosphorylation by kinases that are anchored to the axoneme via RSP3 [acting as A-kinase anchoring proteins (AKAP); Gaillard et al., 2006].

Given the absence of both CP/RS and a functional I1/f motor from Ch neuron cilia, it is highly intriguing that they express a cell-type-specific homolog of WDR78/IC138 (CG13930), as well as MIA subunits and the RSPH3/AKAP homolog. This does not appear to be an evolutionary remnant since knockdown of *CG13930* (IC138) and *CG32392* (RSPH3) each affects Ch neuron function. In addition, Ch neurons express at least one homolog of T/TH complex proteins (CFAP43). Again, this is intriguing because the *Chlamydomonas* T/TH complex is associated with I1/f HCs and is proposed to provide mechanical feedback (Fu et al., 2018). However, T/TH complex is also required for IC138 phosphorylation and interacts with RSP3 (Fu et al., 2018).

Taken together, we propose that in Ch neuron cilia, IC138-RSPH3-MIA-T/TH might represent retention of this key regulatory axis for regulating ODA activity and/or the waveform of ciliary movement (Figure 4B). One caveat of this suggestion is that Ch neurons apparently do not express a homolog of IC140, which is required for assembly of the IC138 subcomplex in *Chlamydomonas*. However, there is evidence that IC138 also binds tubulin directly (Hendrickson et al., 2013). Further investigation would require showing that these proteins indeed localize to the Ch cilium. In *Chlamydomonas* the molecular mechanism of how dynein motor activity is regulated is very uncertain (Porter, 2018). Overall, Ch neuron cilia may provide a useful model for future analysis of this regulatory axis, and excitingly this may provide insight into the molecular mechanism of mechanosensory amplification and frequency selectivity in these neurons.

### Implications for Human Motile Cilia Biology and PCD Research

The disease PCD results from the mutation of any one of at least 40 different human genes encoding diverse components of the ciliary motility machinery (Mitchison and Valente, 2017). The high retention of ciliary motility genes in *Drosophila* is reflected in the fact that the majority of PCD-causative genes have functional *Drosophila* homologs (Table 6). This suggests that despite its specialized motile ciliated cell types, *Drosophila* will continue to be a useful metazoan model for PCD studies. Indeed, transcriptome and genetic analyses in *Drosophila* have recently been particularly useful to identify and validate new DNAAFs (*Zmynd10, Heatr2, Wdr92*) (Moore et al., 2013; Diggle et al., 2014; zur Lage et al., 2018). Whilst human ZMYND10 and HEATR2 are known PCD-causative genes, WDR92 remains highlighted as a PCD candidate gene based on the analysis of its *Drosophila* homolog.

**Table 6:** PCD gene homologs in *Drosophila*.

**Human gene**	***Drosophila* gene**	**Expression summary**	**RNAi phenotype**	
			**Uncoordinated**	**Male infertile**
*ARMC4*	*gudu*	Testis only	nd	nd
*C21orf59*	*CG18675*	Ch + Testis	+	+
*CCDC103*	*CG13202*	Ch + Testis	+	+
*CCDC114*	*CG17083*	Testis only	nd	nd
*CCDC114*	*CG14905*	Ch only	+	–
*CCDC151*	*CG14127*	Ch only	+	–
*CCDC39*	*CG17387*	Ch + Testis	+	+
*CCDC40*	*CG41265*	Ch + Testis	nd	nd
*CCDC65*	*CG30259*	Ch only	+	–
*CCNO*	No ortholog			
*DNAAF1*	*dtr*	Ch + Testis	+	+
*DNAAF2/KTU*	*CG1553*	Widely expressed	+	+
*DNAAF3*	*CG17669*	Ch + Testis	+	+
*DNAH11*	*Dhc93AB*	Ch only	+	–
*DNAH5*	*CG9492*	Ch only	+	–
*DNAH8*	*kl-3*	Testis only	nd	nd
*DNAI1*	*CG9313*	Ch + Testis	+	+
*DNAI2*	*CG6053*	Ch only	+	–
*DNAI2*	*CG10859*	Testis only	nd	nd
*DNAI2*	*CG1571*	Testis only	nd	nd
*DNAJB13*	*Dnaj-1*	Ch + Testis	nd	nd
*DNAL1*	*CG8800*	Ch + Testis	nd	nd
*DNAL1*	*CG10829*	Testis only	nd	nd
*DRC1*	*CG10958*	Ch + Testis	+	+
*DYX1C1*	*CG14921*	Ch + Testis	+	+
*GAS8*	*CG14271*	Ch + Testis	+	+
*HEATR2*	*CG31320*	Ch + Testis	+	+
*HYDIN*	No ortholog			
*LRRC56/ODA8*	*CG14185*	Ch only	nd	nd
*LRRC6*	*tilB*	Ch + Testis	+	+
*NME8*	*CG18130*	Testis only	nd	nd
*RSPH1*	*CG5458*	Testis only	nd	nd
*RSPH3*	*CG32392*	Ch + Testis	nd	nd
*RSPH4A*	*CG3121*	Testis only	nd	nd
*RSPH9*	*CG31803*	Testis only	nd	nd
*SPAG1*	*CG18472*	Ch + Testis	+	+
*TTC25*	*CG13502*	Ch + Testis	nd	nd
*ZMYND10*	*CG11253*	Ch + Testis	+	+

*PCD gene list taken from National Health Service (NHS) gene panel v.2.0. Key to phenotypes: +, impaired; –, unaffected; nd, not determined*.

As shown here, genetically supplied RNAi knockdown in developing sensory neurons (driven by *scaGal4*) and spermatocytes (driven by *BamGal4*) is quite efficient for validating the function of ciliary motility genes in *Drosophila*. RNAi analysis appears particularly effective in showing the function of genes expected to have a major effect on motility (DNAAFs, molecular ruler, etc). RNAi phenotypes as assayed in our study are less consistently clear for other structural proteins, where motility may be less severely affected by the loss of single components. Single-headed IDA HCs may fit this category: knockdown of *Dhc36C* and *Dhc62B* result in partial reduction of fertility and climbing. Similarly, mutation of *CG17150/Dnah3* caused deafness, but fertility appeared normal unless tested by a sperm competition assay (Karak et al., 2015). Of course, it is important to bear in mind that a lack of effect could also reflect incomplete effectiveness of RNAi knockdown for the lines tested rather than a partial requirement in ciliary motility. This could explain unexpectedly negative results of knockdown for some genes (e.g., *CG17564/MIA*). Follow up of specific genes identified in this study will require better characterization of knockdown efficiency, or more likely the generation of specific mutations. Nevertheless, as an initial screening tool transcriptome analysis followed by RNAi analysis is largely effective, and the conservation of ciliary machinery in *Drosophila* is such that it will continue to play a useful role in identification, validation and analysis of PCD genes.

Beyond gene discovery and validation, there is an urgent requirement for mechanistic analysis of known PCD genes. This is particularly true of DNAAFs because the possibility that they are co-chaperones for motor protein folding, stabilization and assembly raises the potential of therapeutic intervention in PCD such as “chaperone therapy.” Indeed this is supported by our recent discovery in *Drosophila* that the Hsp90 co-chaperones, *Wdr92/CG14353* and *Pih1d1/CG5792* cause PCD when mutated in *Drosophila* (zur Lage et al., 2018). Neither gene is yet clearly linked to human PCD but they are strong candidates. In contrast, two other PIH domain proteins are known PCD-causative DNAAFs in humans—*KTU/DNAAF2* (Omran et al., 2008) and *PIH1D3* (Olcese et al., 2017; Paff et al., 2017). *CG5048* is the *Drosophila* ortholog of *PIH1D3*, and is highly expressed in Ch neurons and testes, and its knockdown causes strong motility phenotypes. *nop17l* is the ortholog of *DNAAF2/KTU*. Exceptionally, *nop17l* is not strongly enriched in Ch neurons and whilst expressed moderately in testes, it is also expressed strongly in other adult tissues. *nop17l* may have a broader function beyond dynein assembly in *Drosophila*. It is notable that *DNAAF2/KTU* mutations are a rare cause of PCD, suggesting that most human mutations may not be viable (Omran et al., 2008). Recent analysis in zebrafish suggests that different PIH-domain DNAAFs are required for assembly of different subsets of motors (Yamaguchi et al., 2018). We suggest that *Drosophila* will be a useful model system for understanding the complex and possibly overlapping roles of PIH-domain DNAAFs.

A major implication for PCD research stems from our clear demonstration of strong cell type specificity in dynein motor subunit expression and function. We found in *Drosophila* that dynein motor HCs vary between cell types both by their presence/absence and also in one case by presence of a cell-type specific isoforms. The severity of human PCD is variable in terms not only of the degree of impaired ciliary motility but also of co-presentation of different phenotypes. For instance, the prevalence of situs inversus varies between PCD gene mutations (Olbrich et al., 2015). How much does cell type specificity in motile gene expression/function underlie PCD phenotype variability? Given that the *Drosophila* cell-type-specific ODA HC variants correspond to specific human orthologs it is possible that cell-type-specific functional specialization of ODAs is also found in humans. However, the cell-type specific distribution and function of human dyneins is very poorly known. DNAH17 is expressed in both human and *Drosophila* sperm, but it is not known in humans whether it is restricted to sperm like it is in *Drosophila*. Human DNAH5/8/9/11 are all expressed in both respiratory cilia and sperm (Yagi and Kamiya, 2018). PCD mutations in these HC genes correlate with differences in phenotype and severity, which may reflect quantitative expression differences of these dyneins across cell-types, but so far there is no evidence for complete cell-type specificity (Yagi and Kamiya, 2018).

It is interesting that the three ODA HC genes that have been found to be mutated in PCD (DNAH5, DNAH9 and DNAH11) are orthologous to the three ODA HC genes that function in Ch neurons, despite the highly specialized nature of Ch cilium motility (Kollmar, 2016). This might suggest that Ch neurons may model human motile cilium biology better than *Drosophila* sperm.

The other major structural difference between Ch neuron cilia and sperm flagella is their 9+0 vs. 9+2 axonemal structures, and accordingly Ch neurons do not express most RS subunit genes. The absence from Ch neurons of the IDA I1/f motor complex is consistent with this, since in the *Chlamydomonas* flagellum it is regulated by signals transmitted from CP/RS and N-DRC (Viswanadha et al., 2017). It would therefore be interesting to determine whether the lack of I1/f motor is a feature of 9+0 motile cilia more generally, including the nodal cilia of vertebrate embryos that are required for left-right axis asymmetry.

## Conclusion

*Drosophila* retains almost all ciliary motility machinery, including homologs of almost all known PCD genes. There are notable differences between the two motile ciliated cell types, which might be usefully exploited in the future to further the understanding of ciliary motility. It will continue to be a useful genetic model in the future for validation and functional analysis of ciliary motility genes.

## Data Availability Statement

The microarray datasets generated for this study can be found in the Gene Expression Omnibus repository (accession: E-MTAB-7529).

## Author Contributions

PzL conducted most of the experiments and data analysis, contributed to experimental design, and edited the manuscript. FN carried out *in situ* hybridisations. AJ conducted data analysis, contributed to experimental design, and wrote the manuscript. All authors contributed to manuscript revision, read, and approved the submitted manuscript.

### Conflict of Interest Statement

The authors declare that the research was conducted in the absence of any commercial or financial relationships that could be construed as a potential conflict of interest.
