# Supplementary material for: Survey of the Ciliary Motility Machinery of Drosophila Sperm and Ciliated Mechanosensory Neurons Reveals Unexpected Cell-Type Specific Variations: A Model for Motile Ciliopathies
Source: Front Genet. 2019 Feb 1;10:24. doi: 10.3389/fgene.2019.00024 (PMC6367277; doi:10.3389/fgene.2019.00024)
Supplement: Supplementary file 2 [file Table_2.DOCX]

Table S2. Dynein light chains (LC) in *Drosophila*. Column headings and abbreviations as given for Table 1.

| **Gene** | **Name** | **Class/name** | ***Chlamydomonas*** | **Human** | **Arm** | **Ch expression** | **Testis expression** | **Expression summary** |
| --- | --- | --- | --- | --- | --- | --- | --- | --- |
| *CG8800* |  | LC1/DNAL1 | LC1/DLU1 | *DNAL1* | ODA | 3.28 | 8.4 (84.6) | Ch + Testis |
| *CG10839* |  | LC1/DNAL1 | LC1/DLU1 | *DNAL1* | ODA | -1.15 | 10 (550) | Testis only |
| *CG12363* | *Dlc90F* | Tctex2 | LC9 | *DYNLT1/Tctex1* | ODA, I1/f, cyto, IFT | 1.56 | 1.8 (1123) | Widely expressed |
| *CG7276* |  | Tctex2 | LC2/DLT2 | *TCTEX1D2* | ODA, I1/f | 1.00 | 12 (454) | Testis only |
| *CG5359* |  | Tctex2 | LC2/DLT2 | *TCTEX1D2* | ODA, I1/f | 5.62 | 2.3 (154) | Ch + Testis |
| *CG18130* |  | LC3/5 | LC3/LC5 | *NME8/9/TXNDC3* | ODA | 1.09 | 15 (231) | Testis only |
| *CG15547* |  | LC3/5 | LC3/LC5 | *NME8/9/TXNDC3* | ODA | 1.41 | 8.9 (242.2) | Testis only |
| *CG14221* |  | LC3/5 | LC3/LC5 | *NME8/9/TXNDC3* | ODA | 1.28 | nd | nd |
| *CG10751* | *roadblock* | LC7 | LC7a/b, DLR1/2 | *DYNLRB1/2* | ODA, I1/f, cyto, IFT | 1.43 | 1.1 (557) | Widely expressed |
| *CG34192* | *robls54B* | LC7 | LC7a/b, DLR1/2 | *DYNLRB1/2* | ODA, I1/f, cyto, IFT | 11.56 | 8.2 (15) | Ch + Testis |
| *CG1014* | *robl62A* | LC7 | LC7a/b, DLR1/2 | *DYNLRB1/2* | ODA, I1/f, cyto, IFT | 1.09 | 17 (2018) | Testis only |
| *CG10838* | *robl22E* | LC7 | LC7a/b, DLR1/2 | *DYNLRB1/2* | ODA, I1/f, cyto, IFT | -1.19 | 14 (455) | Testis only |
| *CG15171* | *robl37BC* | LC7 | LC7a/b, DLR1/2 | *DYNLRB1/2* | ODA, I1/f, cyto, IFT | 1.11 | 11 (424) | Testis only |
| *CG10822* |  | LC7 | LC7a/b, DLR1/2 | *DYNLRB1/2* | ODA, I1/f, cyto, IFT | 1.10 | 12 (840) | Testis only |
| *CG10834* |  | LC7 | LC7a/b, DLR1/2 | *DYNLRB1/2* | ODA, I1/f, cyto, IFT | 1.03 | 18 (431) | Testis only |
| *CG16837* |  | LC7 | LC7a/b, DLR1/2 | *DYNLRB1/2* | ODA, I1/f, cyto, IFT | 1.05 | 15 (471) | Testis only |
| *CG6998* | *ctp* | LC8 | LC8 | *DYNLL1* | ODA, I1/f, cyto, IFT | -1.03 | 0.8 (920) | Widely expressed |
| *CG8407* |  | LC10 | LC10 | *DNAL4* | ODA | -1.51 | 14 (434) | Widely expressed |
